# Facilitators and Barriers to Implementing Evidence-Based Clean Intermittent Catheterization After Radical Hysterectomy: A Mixed-Methods Study

**DOI:** 10.3390/jcm15134925

**Published:** 2026-06-24

**Authors:** Lu Xing, Biru Luo, Yuqing Song, Huaping Fu, Wen Zhao, Xue Deng

**Affiliations:** 1 Department of Pelvic Floor Medicine Center Nursing, West China Second University Hospital, Sichuan University, Chengdu 610041, China; 2 Key Laboratory of Birth Defects and Related Diseases of Women and Children (Sichuan University), Ministry of Education, Chengdu 610041, China; 3 Department of Nursing, West China Second University Hospital, Sichuan University, Chengdu 610041, China

**Keywords:** cervical cancer, radical hysterectomy, clean intermittent catheterization, barriers, mixed-methods study

## Abstract

**Objective:** To analyze the perceived facilitators and barriers promoting and hindering the clinical application of the best evidence of clean intermittent catheterization (CIC) in patients after radical hysterectomy (RH). **Methods:** This study employed a convergent parallel mixed-methods design. Participants included patients undergoing CIC after RH, medical and nursing practitioners and managers in the gynecological department and outpatient clinics at a tertiary-level women’s and children’s hospital in Chengdu. They were included in both components separately. Interview data were managed using Nvivo 11.0 software and analyzed through directed content analysis. Quantitative data were analyzed using SPSS 29.0 statistical software. **Results:** A questionnaire survey was conducted among 156 healthcare providers and 300 patients. Qualitative interviews were conducted with 11 healthcare workers and 12 patients. At the evidence itself level, evidence meeting clinical needs and evidence lacking practical applicability, respectively, promoted and hindered clinical implementation of the best evidence. At the potential adopters’ level, healthcare professionals’ insufficient professional competence, low willingness to promote implementation, numerous concerns, and lack of autonomy and awareness regarding the importance of the task were significant barriers, but they maintained an overall positive attitude toward the application. At the practical environment level, patient-related perceived barriers predominantly hindered evidence implementation. Additionally, a supportive practice atmosphere, economic feasibility, and talent development opportunities served as key facilitators. However, existing nursing practice content and workflows directly impacted evidence adoption. **Conclusions:** The promotion and barriers to the clinical application of the best evidence for CIC in RH postoperative patients are multifaceted. Targeted intervention strategies must be developed to facilitate the effective translation of evidence into clinical practice.

## 1. Introduction

Cervical cancer (CC) poses a significant global health threat to women, ranking fourth in both incidence and mortality among female cancers [1]. Radical hysterectomy (RH) stands as the primary and most prevalent surgical intervention for CC [2,3,4]. However, due to factors such as prolonged operation duration and extensive tissue resection, RH may lead to diverse postoperative complications [5,6,7]. Among these, bladder dysfunction emerges as the most prevalent complication following RH and is considered highly distressing for patients [8]; research indicates an incidence rate ranging from 12% to 85% for this complication [9].

Patients undergoing RH surgery typically require long-term indwelling urinary catheters to manage potential postoperative urinary retention [10]. However, this practice can lead to several complications, including pain, catheter-associated urinary tract infections, and urethral injury [11,12]. Consequently, the prompt removal of the urinary catheter following surgery is crucial for preventing these issues.

Clean intermittent catheterization (CIC) is a method allowing non-medical personnel (such as the patient or a family member) to insert a catheter through the urethra into the bladder in a clean setting to regularly empty the bladder. This approach is endorsed by the International Continence Society (ICS) as the safest and most effective technique for aiding patients with neurogenic bladder in bladder emptying, establishing it as the gold standard [13].

However, awareness and adoption of clean intermittent catheterization (CIC) remain low among patients globally. Furthermore, even among those who choose this method, inadequate technique and poor adherence frequently compromise its therapeutic efficacy [14,15,16,17]. Medical professionals worldwide encounter challenges in advocating for and executing CIC, resulting in its restricted adoption [17,18,19]. Our project team had previously synthesized the best evidence on CIC of RH in CC patients, but the challenges and obstacles faced by patients and healthcare providers during CIC were not addressed, and other previous research has also rarely mentioned the barriers that hinder the widespread adoption of CIC in CC patients after RH, which directly contributes to the persistent difficulties in effectively implementing CIC.

This study aimed to employ a mixed research design to comprehensively assess both the perceived facilitators and barriers, and measured levels influencing the clinical utilization of the best evidence of CIC in patients after RH.

## 2. Materials and Methods

The evidence-based CIC recommendations evaluated in this study were derived from a prior evidence summary conducted by the research team. The summary included 9 themes and 40 recommendations covering CIC assessment, CIC method, types of urinary catheters, timing for CIC, CIC frequency, drinking plan, urination diary, health education, continuity of care and follow-up. Evidence quality and recommendation strength were assessed using AGREE II, AMSTAR 2, Cochrane RoB 2.0 and the JBI 2014 Evidence Pre-grading System. Before data collection, the recommendations were reviewed by clinical experts and evidence-based methodology experts, and adapted to the local gynecological oncology setting.

Guided by the Knowledge-to-Action (KTA) framework [20,21], this study employed a convergent parallel mixed-methods design. Quantitative and qualitative data were collected concurrently, analyzed separately, and then integrated at the interpretation stage using a joint-display matrix to identify convergent, complementary, and discrepant findings. The analysis examined perceived facilitators and barriers affecting evidence application from three KTA-informed perspectives: the evidence itself, potential adopters, and the practice environment.

The protocol for this study had been registered with the Fudan University Center for Evidence-Based Nursing under registration number ER20230463.

### 2.1. Qualitative Research

#### 2.1.1. Materials and Patients

A descriptive qualitative research design and maximum-variation purposive sampling within purposive sampling were employed. The study included patients who had undergone CIC following RH at a tertiary Grade A women and children’s specialized hospital between March 2025 and June 2025. The research subjects met the following inclusion criteria: being aged 18 years or older, having undergone RH and CIC at the hospital where the study took place, currently undergoing CIC, absence of cognitive or mental disorders, and proficient communication skills. Exclusion criteria comprised severe organic lesions in organs like the heart, brain, and lungs, water, electrolyte, or acid–base balance disorders, severe kidney disease or bladder and urethral surgery, ongoing urinary tract infection, inability to complete the data collection process, and refusal to participate in the study. In this study, severe kidney disease was defined as meeting any of the following criteria: (1) an estimated glomerular filtration rate (eGFR) < 30 mL/min/1.73 m^2^; (2) serum creatinine > 450 μmol/L; (3) end-stage renal disease requiring hemodialysis, peritoneal dialysis, or kidney transplantation; (4) a documented diagnosis of autosomal dominant or recessive polycystic kidney disease, lupus nephritis, ANCA-associated vasculitis, or other forms of glomerulonephritis with severe renal impairment (e.g., eGFR < 30 mL/min/1.73 m^2^, serum creatinine > 450 μmol/L, or requiring dialysis/transplantation); (5) any other primary or secondary kidney disease that, in the investigator’s judgment, posed a substantial risk to the subject or interfered with study outcomes. Two physicians, each with over 10 years of experience in the urology ward, independently screened the patients. In cases of disagreement, a third physician with similar experience was consulted to reach a consensus.

In addition, the study included medical and nursing practitioners and nursing managers from the gynecology ward and outpatient department between July 2025 and October 2025. The inclusion criteria were: holding a physician or nurse practice certificate; having over 5 years of clinical experience in gynecological medical treatment/nursing, with nursing managers requiring over 10 years of experience; holding a Bachelor’s degree or higher; and voluntarily participating in the study with informed consent. Exclusion criteria comprised visiting, standardized training and intern doctors/nurses in gynecological wards or outpatient departments, as well as those not on duty during the research period.

#### 2.1.2. Sample Size

Consistent with the fundamental principles of qualitative research, the sample size and richness of the data were determined by the criterion of data saturation. When no new themes emerged from the interview content, three additional participants were recruited. If no new information was identified after data analysis, saturation was considered to have been achieved, and further participant recruitment was stopped [22].

#### 2.1.3. Interview Guide

The preliminary interview guide was developed based on the Ottawa Model of Research Use (OMRU) [23,24,25] and the FAME framework [26], informed by a literature review. The initial draft was then reviewed and revised by two experts in evidence-based methodology and nursing. Their feedback was incorporated through team discussions, and modifications were made to enhance the clarity, relevance, and comprehensiveness of the questions.

Subsequently, the revised guide was tested in pre-interviews with two patients and two medical staff members to verify whether the questions accurately and comprehensively captured the necessary information. After each pre-interview, the research team debriefed and systematically documented participants’ comments regarding question wording, sequence, and understandability. All suggestions were discussed among the team, and consensus was reached on necessary adjustments. The guide was then modified accordingly. This iterative process continued until no further issues were identified, resulting in the final version of the interview guide (presented in Table 1).

#### 2.1.4. Data Collection

Interviews were conducted in a quiet, private setting [27]. Prior to data collection, the researcher contacted the head of the relevant department to provide a detailed overview of the research plan and its significance. After obtaining approval, the department head identified eligible interviewees based on predefined criteria. Information about medical staff and patient participants was gathered through the department head and the designated nurse, respectively.

On the day of the interview, the researcher arrived punctually at the interview site with the necessary equipment. Before starting, the researcher explained the objectives, importance, and procedures of the study to each participant in detail. Written informed consent was obtained from all participants prior to any interview arrangements. Specific interview times and locations were scheduled only after the consent form was signed.

At the beginning of each interview, the researcher re-explained the purpose and significance of the study and activated the recording device (iFLYTEK X3 Pro, iFLYTEK, Hefei, China) only after obtaining verbal consent again. The researcher then conducted face-to-face discussions following a semi-structured interview outline, while simultaneously taking notes. Each interview session lasted approximately 30–40 min for patients and 30–60 min for medical staff.

#### 2.1.5. Qualitative Data Analysis

The researchers compiled interview content in real-time and completed audio transcription within 48 h. Once all transcribed texts were uniformly numbered, data management utilized Nvivo 11.0 software, with researchers responsible for data archiving. Data analysis employed the directional content analysis method, including holistic cognitive data analysis, construction of a classification matrix (Table 2), manual encoding and classification, identifying meaning units and classifying them into a predefined matrix, logging in to the information again, category establishment and theme refinement. Following this, sub-themes were compared, integrated, and the core theme was ultimately derived.

#### 2.1.6. Rigor and Trustworthiness

Two researchers independently coded the transcripts using a directed content-analysis approach. Discrepancies were resolved through discussion with a third researcher. An audit trail was maintained throughout coding. Data saturation was assessed separately for patients and healthcare professionals. Representative quotations were translated from Chinese into English and back-translated by a bilingual researcher to preserve meaning.

### 2.2. Quantitative Research

#### 2.2.1. Materials and Participants

A cross-sectional research design was employed, utilizing convenience sampling. Patients who had undergone CIC following RH and had been treated at a tertiary grade A women and children’s specialized hospital between March 2025 and October 2025 were selected as the study participants. The eligibility criteria for patients mirrored those of qualitative studies. Additionally, medical and nursing workers, along with nursing managers from the gynecological wards and outpatient departments of the same hospital between May 2025 and June 2025, were included in the study. Inclusion criteria require at least one year of clinical experience in gynecological medical care or nursing practice, and all other inclusion and exclusion criteria are identical to those for qualitative research.

#### 2.2.2. Sample Size

The sample size of patients was calculated using the standard formula for continuous variables: $n={\left(\frac{Z_{\alpha /2}S}{\delta }\right)}^{2}$. In this formula, S represents the sample standard deviation, δ represents the allowable error, and $Z_{\alpha /2}$ is the bilateral Z-value at a specified significance level. A two-sided test was adopted with α = 0.05, corresponding to $Z_{\alpha /2}=1.96$, and the allowable error was set at no more than 5 points. A pilot survey was conducted among 50 patients using the CIC Self-Management Questionnaire. The mean self-management score was 131.07 ± 28.12. Based on these data, the required sample size was calculated to be 122 patients. Accounting for a potential dropout rate of 20%, the final sample size was determined to be at least 146 patients.

For healthcare providers, given the exploratory nature of the survey and the fact that we aimed to capture perspectives from all available staff who met the inclusion criteria, we adopted a convenience sampling approach and invited all eligible staff across multiple departments to participate.

#### 2.2.3. Measurements

##### General Information Survey

The researchers developed a general information questionnaire based on the literature review. The patient version of the scale encompassed demographic details and disease-specific data, including age, height, weight, occupation, educational level, CC stage, and disease progression. The medical staff version of the scale comprised demographic factors like age, highest level of education, years of professional experience, and job-related particulars.

##### Clean Intermittent Self-Catheterization of Knowledge, Attitude, Practice Scale (CISC-KAPS)

The scale was developed by Chen et al. [28] in 2021 and includes 19 items across three dimensions (knowledge, attitude, and behavior). A 5-point Likert scale is used, with some items reverse-scored. Knowledge items are scored from 1 (“completely unaware”) to 5 (“completely aware”); attitude items from 1 (“strongly disagree”) to 5 (“strongly agree”); and behavior items from 1 (“never”) to 5 (“often”). Total scores range from 19 to 95, with higher scores indicating higher levels of knowledge, attitude, and behavior. Item wording was adapted when administered to healthcare providers. The scale had a content validity index of 0.888, an explained variance of 63.275% from three factors in exploratory factor analysis, and an overall Cronbach’s α of 0.937 [28].

##### CIC Acceptance Test (ICAT)

The Chinese version of the ICAT was translated by Wang et al. [29] in 2021. The scale consists of 14 items distributed across three dimensions: fear, self-esteem, and acceptance. A 5-point Likert scale is used, where 0 = strongly disagree, 1 = disagree, 2 = neutral, 3 = agree, and 4 = strongly agree. Patients’ acceptance levels are assessed by calculating the total score and the subscale scores, with higher scores indicating lower levels of acceptance. Total scores range from 0 to 56. The Chinese version demonstrated a content validity coefficient of 0.932, an overall Cronbach’s α of 0.805, and subscale Cronbach’s α values ranging from 0.736 to 0.875 [29].

##### CIC Difficulty Questionnaire (ICDQ)

The ICDQ was developed by Guinet-Lacoste et al. in 2016 [30], and its Chinese version was used in this study [31]. The scale has 13 items, each rated on frequency and degree using a 4-point Likert scale (frequency: 0 = never to 3 = always; degree: 0 = none to 3 = severe). The mean score per dimension reflects the frequency and level of CIC-related difficulties, with higher scores indicating more frequent and severe difficulties. The Chinese version had a content validity index of 0.98 and a Cronbach’s α of 0.904 [31].

##### CIC Adherence Scale (ICAS)

The scale was developed by Guinet-Lacoste et al. [32] in 2018 and consists of eight items. For the first seven items, each item is scored as 1 for “yes” and 0 for “no,” except for item 5, which is reverse-scored. The eighth item uses a 5-point Likert scale. The total score ranges from 0 to 8 points. A total score of 3–8 indicates poor adherence, 1–2 indicates moderate adherence, and 0 indicates strong adherence. The scale demonstrated a Cronbach’s α coefficient of 0.73 [32].

##### Self-Confidence Scale for Clean Intermittent Self-Catheterization (SCSCISC)

Developed by Biaziolo et al. [33] in 2017, the instrument comprised 16 items and employed a 5-point Likert scoring system. The scoring options ranged from “very unconfident” (1 point) to “completely confident” (5 points), yielding a total score range of 16 to 80 points. The score positively correlated with the patient’s confidence level. The overall Cronbach’s coefficient of the scale achieved 0.944 [33].

##### CIC Self-Management Questionnaire

A self-management scale for patients undergoing intermittent catheterization was developed by Zhang et al. [34] in 2023. The scale comprises 37 items distributed across four domains: disease symptom management, intermittent catheterization behavior management, daily living activities management, emotional management, and social reintegration. A 5-point Likert scale is employed, with scores ranging from 1 to 5, corresponding to “never,” “rarely,” “occasionally,” “sometimes,” and “always,”, respectively. Reverse-coded items are scored in the opposite direction. The total scale score and subscale scores are summed to evaluate the level of self-management; higher scores reflect better self-management. The content validity index for the total scale was 0.939, the overall Cronbach’s α was 0.969, and the subscale Cronbach’s α values ranged from 0.755 to 0.937 [34].

A quantitative investigation was conducted to explore the measured levels of both the patient and medical staff levels. At the patient level, research tools included the General Information Questionnaire, CISC-KAPS, ICDQ, ICAT, ICAS, SCSCISC, and the CIC Self-Management Questionnaire. The medical staff utilized the General Information Questionnaire and CISC-KAPS for their investigation.

#### 2.2.4. Data Collection

On-site data collection occurred through the administration of questionnaires. Patient questionnaires were gathered during visits to the gynecological ward and the CIC clinic, while those from the medical staff were collected within the department during non-working hours. Prior to distributing the questionnaires, the researcher explained the purpose and process of the study in detail to the participants. After all participants signed the informed consent form, they independently completed the questionnaire in a quiet environment. Completing the questionnaire took approximately 15 min. Throughout the process, standardized instructions were employed to ensure data quality.

#### 2.2.5. Statistical Analyses

Two individuals organized, inputted, and managed the data using Epidata 4.0 and conducted the analysis with SPSS 29.0 (IBM Corp., Armonk, NY, USA) statistical software. Statistical significance was defined as a *p* value < 0.05 (two-tailed). Categorical variables were depicted using frequency and percentage. Continuous variables were handled based on their distribution: normally distributed data were described as mean ± standard deviation, and non-normally distributed data were presented as median (interquartile range).

## 3. Results

### 3.1. Participants Characteristics

A questionnaire survey in this study involved 156 medical staff (the actual number of eligible staff across multiple departments) and 300 patients. Table 3 and Table 4 provide detailed general information about the respondents. Qualitative interviews included 11 medical staff and 12 patients. The medical staff’s interviews ranged from 32 to 78 min, while the patients’ interviews ranged from 26 to 45 min. Table 5 and Table 6 present comprehensive information about the interviewees.

### 3.2. Evidence Application Facilitators/Barriers

This phase employs the KTA framework as the theoretical foundation, conducting an in-depth analysis of perceived facilitators and barriers, and measured levels affecting evidence application from three perspectives: the evidence itself, potential adopters, and the practical environment.

#### 3.2.1. Evidence Itself

The findings regarding perceived facilitators and barriers influencing evidence utilization at the evidence itself level primarily stemmed from qualitative interviews, mainly including “Limited operational clarity and usability of evidence” and “Evidence meets clinical needs”, as detailed in Table 7.

#### 3.2.2. Potential Adopters

The potential adopters in this study refer to healthcare personnel at the pilot conversion bases. The analysis of perceived barriers/facilitators and measured levels at this level was derived from both qualitative interviews and questionnaire surveys.

##### Qualitative Research of Perceived Facilitators/Barriers

The main perceived barriers were “Insufficient knowledge and skills”, “Low willingness among healthcare staff to promote and adopt the practice”, “Insufficient autonomy in practice”, “Lack of recognition regarding task importance”, “Healthcare workers’ concerns” and “Nurse shortage”, but “Positive attitude toward evidence application” was the perceived facilitators, as shown in Table 8.

##### Quantitative Research of Measured Levels

Suboptimal knowledge, attitudes, and behaviors among healthcare staff: Specific scoring details were presented in Table 9. While the mean scores for all items in the knowledge dimension exceeded the average, the mean scores for items in both the attitude and behavior dimensions fell below the average.

#### 3.2.3. Practical Environment

The findings regarding the perceived facilitators/barriers and measured levels at the environmental level in this study were derived from both qualitative interviews and questionnaire surveys.

##### Qualitative Research of Perceived Facilitators/Barriers

The details on perceived barriers/facilitators are shown in Table 10. All the sub-themes of the “Current nursing practice content and processes” and “Practical atmosphere” were perceived barriers and facilitator, separately. Sub-themes of “Talent development” were all the perceived facilitators. Except for “Social support” and “Image and comfort requirements”, other sub-themes of the “Patient factors” were perceived barriers.

##### Quantitative Research of Measured Levels

Suboptimal levels of patient knowledge, attitudes, and behaviors: Specific scores are shown in Table 11. Under the condition that each item is scored out of 5 points, only the average score for the knowledge dimension exceeded 3 points, while the average scores for both the attitude and behavior dimensions remained below 3 points.

Inadequate patient acceptance of CIC: See Table 12 for detailed scores.

Significant difficulties in implementing CIC for patients: Scores for both the frequency and severity dimensions of these difficulties exceeded the average, as shown in Table 13.

Poor patient adherence to CIC: The total score was 3.39 ± 1.19. The top three entries, sorted by average score from highest to lowest, were: “Have you ever reduced the frequency of self-catheterization or stopped it entirely without consulting your doctor because you found it uncomfortable?” “When symptoms lessen or disappear, do you sometimes stop or reduce the frequency of self-catheterization?” and “Having to self-catheterize daily can be experienced as a real burden by some people. Is your self-catheterization routine and practice at times upsetting for you?”

Moderate patient confidence in performing CIC: The total scale score was 55.25 ± 13.50. The top three items ranked from lowest to highest scores were: “I know how to handle blood in urine,” “I can correctly insert a urinary catheter,” and “I know how to manage when there is no urine output.”

Patient CIC-related self-management levels were inadequate: Total self-management scores and scores for each dimension were detailed in Table 14.

### 3.3. Joint Display Integrating Qualitative and Quantitative Findings

The results of qualitative and quantitative studies were analyzed through an integrated analysis that examined three dimensions: evidence itself, potential adopters, and practical environment. The results of this integrated analysis were categorized into two types, as shown in Table 15: convergence and divergence.

## 4. Discussion

By integrating three perspectives of the KTA framework—evidence itself, potential adopters, and the practical environment—we identified two core themes. First, barriers at both the patient and healthcare provider levels were particularly prominent, suggesting that preparedness at a single level alone is insufficient to ensure successful implementation of evidence-based practice without targeted support for the other level. Second, environmental factors, including a lack of structured follow-up and limited educational resources, exacerbated individual-level challenges, creating a vicious cycle in which low confidence and poor CIC outcomes reinforce each other. These findings underscore the need for multi-level interventions that encompass not only healthcare provider training but also patient empowerment and systemic restructuring.

### 4.1. Discussion of Perceived Barriers/Facilitators from the Evidence Itself

Regarding the evidence itself, we identified both perceived barriers and facilitators. The fact that the evidence addressed the clinical needs of both medical staff (as evidence users) and patients (as evidence recipients) served as a key promoter. This alignment filled a gap in CIC nursing management after radical hysterectomy in our unit and provided an intrinsic motivation for evidence adoption [35]. In contrast, the limited feasibility of some evidence—specifically unclear wording and complex content—hindered its clinical application, a finding consistent with Guan et al. [36]. Taken together, these observations suggest a clear takeaway: future evidence translation efforts should prioritize simplifying the content of evidence and presenting it in more accessible formats, such as visualizations, to reduce reliance on dense text. Moreover, the complexity of evidence disproportionately affects patients and frontline providers, linking this evidence-level barrier directly to the challenges of the level of potential adopters and the practical environment level discussed below.

### 4.2. Discussion of Perceived Barriers/Facilitators and Measured Levels from the Potential Adopters

At the potential adopter level, several barriers related to healthcare providers emerged. Consistent with Zhao et al. [37], we found that inadequate knowledge and skills regarding CIC and evidence-based practice-identified in both quantitative and qualitative data-directly undermined effective evidence application. Moreover, a lack of job autonomy and a perceived low importance of the task further reduced motivation and sense of responsibility among medical staff [38]. Importantly, these provider-level barriers did not exist in isolation. The limited feasibility and complex wording of some evidence (discussed above) likely contributed to the knowledge gaps observed, while the absence of structured follow-up and educational resources (environmental factors) may have exacerbated the perceived lack of autonomy. Despite these obstacles, medical staff in our study demonstrated a generally positive attitude and willingness to support evidence implementation-a finding that aligns with the knowledge-attitude-practice theory, where attitude plays a pivotal role in shaping behavior [39]. The key takeaway is that future interventions must simultaneously address knowledge deficits (e.g., through targeted training), structural constraints (e.g., by enhancing job autonomy and clarifying task importance), and attitudinal reinforcement. Drawing on self-efficacy theory and the knowledge–attitude–practice model, strategies such as illustrating positive and negative outcomes of CIC evidence application could help sustain and strengthen the existing positive attitude [40].

### 4.3. Discussion of Perceived Barriers/Facilitators and Measured Levels from the Practical Environment

At the practical environment level, multiple perceived barriers and facilitators, and measured levels were revealed during evidence implementation. On the patient side, barriers included inadequate knowledge, attitudes, and behaviors regarding CIC, as well as low acceptance, adherence, confidence, and self-management, compounded by implementation difficulties and limited patient education. Lack of leadership endorsement, insufficient interdisciplinary collaboration, and the absence of a continuous learning culture impeded progress [41]. Economic perceived factors—affecting staff earnings, patient costs, and implementation-related expenditures—further undermined evidence utilization. Importantly, these environmental barriers did not operate in isolation. They directly exacerbated the individual-level barriers among patients and providers described above. For example, limited health education and unstructured follow-up contributed to patients’ low confidence and poor adherence, while lack of organizational support amplified providers’ perceived low job autonomy. Despite these obstacles, several environmental factors emerged as perceived promoters. Talent cultivation approaches—including training programs, job setting and management, and career development for nurses—significantly enhanced staff motivation [42].

Current CIC nursing practices (e.g., management norms, work processes, health education, and follow-up) remain areas in need of optimization. For example, although videos and brochures are currently available, patient education has not achieved the desired outcomes, largely due to the absence of ongoing monitoring and evaluation mechanisms. At present, medical and legal risks associated with intermittent catheterization are major concerns for healthcare providers, and complex documentation requirements have become a significant challenge. Therefore, it is essential to establish implementation procedures that ensure risk control and streamline documentation. Furthermore, physicians play a crucial role in patient acceptance. As evidenced by our interview findings, patients are often more inclined to follow the professional recommendations of their physicians than those of nursing staff or family members. The key takeaway is that successful evidence implementation requires a supportive environment that addresses both patient-level deficits and system-level deficiencies. Future interventions should prioritize redesigning care pathways and embedding continuous training mechanisms into routine practice. In parallel, we have been actively engaging with local health insurance authorities to seek reimbursement policies for intermittent catheterization supplies, aiming to address cost as a structural barrier.

### 4.4. Limitation

This study has several limitations. First, all interviews and surveys of healthcare providers and patients were carried out at a single tertiary Grade A hospital, which may limit the generalizability of the findings. Future multicenter studies are warranted to enhance the generalizability. Second, convenience sampling was used for the quantitative component, introducing selection bias. Third, the survey relied on self-reported measures, which may be affected by recall and social-desirability bias.

In addition, to enhance the robustness and significance of future findings, it is recommended that subsequent studies include three patient groups: independent voiding, indwelling catheter and CIC. This would allow exploration of differences in patient perspectives across different urinary management strategies and capture patients’ views before initiating intermittent catheterization.

## 5. Conclusions

Using the KTA framework, this mixed-methods study identified barriers and facilitators to implementing evidence-based CIC recommendations among post-radical hysterectomy patients. Key barriers included limited operational clarity of the evidence, insufficient CIC-related knowledge and skills among healthcare professionals, patient concerns, low confidence, poor adherence, inadequate follow-up, and limited institutional resources. Future work should develop and test a structured implementation strategy that includes standardized workflows, staff training, patient education tools, follow-up mechanisms, physician engagement, and reimbursement support.

## Figures and Tables

**Table 1:** Qualitative research interview guide.

Patients with CIC After RH	Healthcare Professionals
1. How did you learn about CIC? When did you become aware of it?	1. How applicable do you think the evidence-based CIC recommendations is in your unit? Why?
2. If you were asked to follow the evidence-based CIC recommendations to perform CIC, would you be willing? Why?	2. How feasible do you think it is to implement evidence-based CIC recommendations in your unit? Why?
3. How do your family members view CIC? Why?	3. To what extent do you accept implementing evidence-based CIC recommendations in your unit? Why?
4. What factors do you think helped you adopt evidence-based CIC recommendations to perform CIC?	4. Please elaborate on the potential barriers to implementing evidence-based CIC recommendations in your unit.
5. What factors do you think hindered you from adopting evidence-based CIC recommendations to perform CIC?	5. Can you propose methods or strategies to overcome the above barriers?
6. What kind of support do you need to carry out CIC according to evidence-based CIC recommendations?	6. What factors do you think would facilitate the implementation of evidence-based CIC recommendations in your unit?
7. Is there anything else you would like to add?	7. What support do you need to use evidence-based CIC recommendations?
	8. Is there anything else you would like to add?

CIC: Clean Intermittent Catheterization; RH: Radical Hysterectomy.

**Table 2:** Summary of classification matrix.

Primitive Category	Operational Definition	Initial Coding
Evidence Itself	Professionals translate research findings into practical tools tailored to specific contexts, continually refining and adapting these findings to facilitate the clinical translation of evidence	Feasibility Applicability Effectiveness Acceptability
Potential adopters	Healthcare workers adopting and applying evidence	Knowledge Skills Attitude Habits/Preferences Current status Concerns
Practical Environment	The physical and social environment in which healthcare workers conduct clinical practice	Perceived patient facilitators and barriers Perceived economic facilitators and barriers Perceived cultural facilitators and barriers Perceived organizational facilitators and barriers current status Perceived other uncontrollable facilitators and barriers

**Table 3:** General information for healthcare staff in quantitative study (*n* = 156).

Variable	Category	N (%)/Mean ± SD
Age (mean ± SD, years)	/	39.3 ± 4.43
Ethnicity (n (%))	Han	154 (98.8)
	Tibetan	0 (0)
	Hui	1 (0.6)
	Yi	0 (0)
	Other	1 (0.6)
Highest education level (n (%))	Primary school or below	0 (0)
	Middle school	0 (0)
	High school or technical	0 (0)
	Associate degree	15 (9.6)
	Bachelor’s degree or above	141 (90.4)
Professional title (n (%))	Junior	62 (39.8)
	Intermediate	74 (47.4)
	Associate senior	12 (7.7)
	Senior	8 (5.1)
Years of work experience (n (%))	<10 years	58 (37.2)
	10–19 years	66 (42.3)
	20–29 years	24 (15.4)
	≥30 years	8 (5.1)
Years worked at pilot transformation base (n (%))	<10 years	67 (43.0)
	10–19 years	57 (36.5)
	20–29 years	25 (16.0)
	≥30 years	7 (4.5)
Prior research experience (n (%))	Yes	111 (71.1)
	No	45 (28.9)
Attention to neurogenic bladder management (n (%))	Yes	66 (42.3)
	No	90 (57.7)

SD: Standard Deviation.

**Table 4:** General information for patients in quantitative study (*n* = 300).

Variable	Category	N (%)/Mean ± SD
Age (mean ± SD, years)	/	47.8 ± 3.73
Height (mean ± SD, cm)	/	159.3 ± 4.85
Weight (mean ± SD, kg)	/	56.3 ± 4.45
Ethnicity (n (%))	Han	290 (96.7)
	Tibetan	6 (2.0)
	Hui	0 (0)
	Yi	3 (1.0)
	Other	1 (0.3)
Education level (n (%))	Primary school or below	26 (8.7)
	Middle school	45 (15.0)
	High school or technical	46 (15.3)
	Associate degree	102 (34.0)
	Bachelor’s degree or above	81 (27.0)
Caregiver’s education level (n (%))	Primary school or below	18 (6.0)
	Middle school	48 (16.0)
	High school or technical	55 (18.3)
	Associate degree	88 (29.3)
	Bachelor’s degree or above	91 (30.4)
Caregiver (n (%))	Spouse	186 (62.0)
	Child	54 (18.0)
	Parent	49 (16.3)
	Other	11 (3.7)
Marital status (n (%))	Unmarried	77 (25.7)
	Married	203 (67.6)
	Divorced	14 (4.7)
	Widowed	6 (2.0)
Place of residence (n (%))	Rural	88 (29.3)
	Urban	212 (70.7)
Occupation (n (%))	Student	3 (1.0)
	Government or public sector	38 (12.7)
	Enterprise employee	75 (25.0)
	Self-employed	65 (21.7)
	Retired	97 (32.3)
	Unemployed	22 (7.3)
	Other	0 (0)
Monthly household income per capita (RMB) (n (%))	≤3000	186 (62.0)
	3001–5000	67 (22.4)
	5001–10,000	43 (14.3)
	>10,000	4 (1.3)
Health insurance type (n (%))	None	2 (0.6)
	Provincial/city insurance	148 (49.4)
	Commercial insurance	72 (24.0)
	New rural cooperative	78 (26.0)
	Other	0 (0)
History of neoadjuvant chemotherapy for cervical cancer (n (%))	No	164 (54.7)
	Yes	136 (45.3)
History of radiotherapy for cervical cancer (n (%))	No	118 (39.3)
	Yes	182 (60.7)
Prior CIC experience (n (%))	No	282 (94.0)
	Yes	18 (6.0)
Catheter replacement before first CIC (n (%))	No	75 (25.0)
	Yes	225 (75.0)
Postoperative infection (n (%))	No	234 (78.0)
	Yes	66 (22.0)
Pathological type (n (%))	Squamous cell carcinoma	228 (76.0)
	Adenocarcinoma	64 (21.3)
	Adenosquamous carcinoma	8 (2.7)
	Other	0 (0)
Tumor stage (n (%))	Stage I	154 (51.4)
	Stage II	112 (37.3)
	Stage III	34 (11.3)
	Stage IV	0 (0)

SD: Standard Deviation.

**Table 5:** General information for healthcare staff in qualitative study (*n* = 11).

ID	Age (Years)	Gender	Education	Professional Title	Position	Work Experience (Years)	Specialty Area	Interview Duration
M1	44	Female	Master	Associate senior nurse	Head nurse	18	Nursing management	78 min 11 s
M2	38	Female	Master	Associate senior nurse	Head nurse	11	Gynecological nursing/management	52 min 33 s
M3	35	Female	Master	Senior nurse	Deputy head nurse	14	Gynecological nursing/management	61 min 18 s
M4	55	Male	Master	Associate senior physician	Deputy director/ward director	31	Gynecological medicine	32 min 44 s
M5	38	Female	Doctor	Attending physician	None	10	Gynecological medicine	41 min 12 s
M6	36	Female	Master	Senior nurse	Nursing team leader	16	Gynecological nursing	44 min 53 s
M7	30	Female	Bachelor	Senior nurse	None	8	Gynecological nursing	50 min 22 s
M8	46	Female	Bachelor	Senior nurse	Nursing team leader	26	Gynecological nursing/bladder management	72 min 56 s
M9	42	Female	Bachelor	Senior nurse	None	21	Gynecological nursing/bladder management	38 min 40 s
M10	40	Female	Master	Senior nurse	Head nurse	15	Obstetrics and gynecology outpatient nursing	48 min 45 s
M11	33	Female	Doctor	Senior nurse	Research nurse	6	Obstetrics and gynecology outpatient nursing	41 min 23 s

**Table 6:** General information for patients in qualitative study (*n* = 12).

ID	Age (Years)	Ethnicity	Education Level	Occupation	Marital Status	CIC Duration (Days)	Caregiver	Interview Duration
P1	54	Han	High school or technical	Self-employed	Married	33	Husband	33 min 17 s
P2	38	Han	Associate degree	Enterprise employee	Married	8	Husband	41 min 26 s
P3	62	Han	Bachelor’s or above	Retired	Married	17	Husband	45 min 48 s
P4	44	Han	Bachelor’s or above	Enterprise employee	Divorced	47	Sister	30 min 12 s
P5	47	Han	Associate degree	Enterprise employee	Divorced	15	Daughter	42 min 57 s
P6	51	Han	Associate degree	Technical professional	Married	62	Husband	44 min 32 s
P7	29	Han	Bachelor’s or above	Enterprise employee	Unmarried	5	Mother	26 min 38 s
P8	48	Tibetan	Bachelor’s or above	Self-employed	Married	3	Husband	28 min 11 s
P9	37	Han	Bachelor’s or above	Technical professional	Married	25	Husband	38 min 33 s
P10	55	Han	Associate degree	Retired	Widowed	34	Son	38 min 52 s
P11	54	Han	Middle school	Self-employed	Married	20	Husband	35 min 37 s
P12	60	Han	High school or technical	Retired	Married	11	Husband	37 min 50 s

CIC: Clean Intermittent Catheterization.

**Table 7:** Qualitative interview findings on perceived facilitators and barriers at the level of the evidence itself.

Theme	Sub-Theme	Facilitator/Barrier	Statements
Limited operational clarity and usability of evidence	/	Perceived barriers: Difficulties exist in directly applying some evidence in clinical practice	M7: The evidence you’ve developed is quite good, but I find it challenging to directly use this evidence to guide my patients. It’s not very clear, and most patients probably wouldn’t understand what this evidence is saying. We need clearer instructions on what to do and how to do it. M11: This content is way too extensive and complex. The descriptions are overly detailed, filled with text that requires time to read and learn. Doesn’t this just increase our workload?
Evidence meets clinical needs	Meeting patient needs	Perceived facilitators: Meeting patients’ needs is a perceived facilitators that affects the application of evidence	P3: I feel like I’m not really clear on this myself. I just started without fully understanding it. When I encounter something I don’t know, I look it up online, but I’m not sure if I can trust the information. P8: What we’re learning now is too fragmented. We learn a little and do a little, but there’s no one to ask. I really want to learn systematically, otherwise the risks are still too high.
	Meeting healthcare workers’ needs	Perceived facilitators: Evidence demonstrates strong clinical applicability, fills gaps in practice, and healthcare providers are willing to adopt it	M5: It’s truly wonderful to have this kind of evidence. Sometimes patients just can’t get their urinary catheters out, and it’s really distressing to see them struggle like that. If we can make this work so they can remove the catheter smoothly, that would be fantastic. M8: I think this evidence is essential to put into practice. I really need something like this when I’m in outpatient clinics. Right now, our explanations to patients are too vague. This evidence is scientifically sound… Plus, even though we’re already doing CIC, our management isn’t very good. I really want a scientific protocol to properly manage this area.

CIC: Clean Intermittent Catheterization.

**Table 8:** Qualitative interview findings on perceived barriers/facilitators at the level of potential adopters.

Theme	Sub-Theme	Facilitator/Barrier	Statements
Insufficient knowledge and skills	Limited CIC knowledge base	Perceived barrier: Lack of relevant knowledge makes it difficult to guide patients.	M6: Many young teachers are largely unaware of what CIC entails, having never encountered it before, and training in this area is also relatively scarce. M9: I’ve actually been performing this procedure quite frequently, but to be honest, I don’t feel like I’ve fully mastered it yet. There’s just too little training available on CIC, and I rarely get the chance to attend specialized courses on this topic. There are still many aspects I’m not entirely clear on.
	Limited evidence-based practice knowledge	Perceived barrier: Limited understanding of evidence-based practice and lack of standardized application capabilities	M7: We may have limited exposure to evidence-based practices. While we often hear about evidence-based approaches, we rarely adhere to them strictly. Faculty with graduate degrees likely have a clearer understanding of this. M9: Evidence-based practice is usually handled by the head nurse and research nurses. We typically just review guidelines and articles during reading reports, rounds, or lectures, then apply the findings—that probably isn’t true evidence-based practice.
	Insufficient CIC-related skills	Perceived barriers: Lack of CIC operational experience and skill training	M3: I’ve been working for over a decade, yet I’ve performed CIC fewer than ten times. Junior nurses likely haven’t done it at all. We rarely receive training for this procedure, and many nurses probably don’t even know how to perform it. M4: We know about CIC, but it’s always done by the nurse instructors. We haven’t performed it ourselves.
Low willingness among healthcare staff to promote and adopt the practice	/	Perceived barriers: Some healthcare staff have doubts about the efficacy of CIC, lack sufficient understanding, or hold reservations, making them reluctant to actively promote its use	M2: Many doctors and nurses aren’t very familiar with CIC. They’re more accustomed to indwelling catheterization in their daily work. For them, it’s a new concept, and they’re unclear about its effectiveness. They might not fully endorse the procedure yet and are hesitant to initiate it for their patients. M4: I know about CIC. Sometimes when our patients can’t have their urinary catheters removed, we also have them do CIC. But its efficacy is still somewhat controversial. Currently, it seems the nursing instructors aren’t performing it very standardizedly either, so it hasn’t been widely adopted. M10: “We don’t have detailed knowledge about CIC’s specifics or outcomes either—just a general understanding. Maybe the outpatient clinic staff aren’t eager to recommend it right away. Currently, most cervical cancer patients only try CIC when indwelling catheters can’t be removed.” M11: “Looking at your materials, CIC seems quite promising.” But I might not be fully informed—can’t indwelling catheters achieve similar results for many patients now?” M1: “patients inserting catheters themselves? Could they learn that? Wouldn’t urinary tract infections become a risk? It seems rather challenging.”
Insufficient autonomy in practice	/	Perceived barriers: Healthcare staff face limited decision-making autonomy due to influences from physicians, supervisors, multidisciplinary teams, and patients during implementation	M2: We genuinely want to implement this, but whether patients agree to CIC largely depends on how the physician communicates it. If the doctor wants the patient to use CIC, mentioning it upfront is far more effective than us trying to persuade them later. It would make our implementation much smoother. M8: This isn’t something I can just decide to implement for patients. After doing it for so long, I’ve realized it requires support from multiple parties—it’s not something any single nurse can manage alone. We need doctors, leadership, multidisciplinary teams, and others involved.
Lack of recognition regarding task importance	/	Perceived barriers: Some healthcare providers underestimate the necessity and urgency of implementing CIC, believing existing practices already meet clinical needs	M5: We’ve had so many patients on indwelling catheters before. It seems many successfully had their catheters removed without urinary complications. We can wait until removal becomes impossible before considering next steps. M7: Do we absolutely have to implement CIC? Patients can remove their catheters without it. Why go through all this trouble teaching patients this new procedure? M11: Is CIC mandatory? Indwelling catheters seem more convenient for patients—no need for multiple daily self-catheterizations or monitoring fluid intake. Would patients even agree to this?
Healthcare workers’ concerns	Perceived risk and liability concerns	Perceived barriers: Concerns over patient self-operation risks, legal liabilities, and medical safety issues	M1: Entrusting everything to patients to operate themselves carries significant risk. Patient training must be rigorously controlled; otherwise, adverse outcomes are highly likely. M2: We definitely need doctors to assess patients first. Only eligible patients should be guided through self-management. After all, many patients have multiple comorbidities, and we can’t predict how smoothly the process will go. Otherwise, there’s still risk involved. M8: Currently, many patient-guided procedures are implemented without proper documentation. There are no medical records or legal safeguards to reference. If complications arise during the process, it’s difficult to refer patients for treatment. We’re very afraid something might go wrong.
	Increased workload	Perceived barriers: Concerns about adding to outpatient and clinical duties, impacting efficiency	M9: Meeting your requirements would definitely require dedicated time. If it takes too long, with so many patients in the clinic every day, we’d never finish work. Patients waiting longer might also voice complaints. M6: Our daily clinical workload is already overwhelming. Adding this interventional guidance—even if not required daily—would still demand time whenever cases arise. I believe assigning dedicated instructors, like for hyperperfusion studies, or referring such patients to specialized interventional clinics, would yield better results. That way, we wouldn’t have to rush through it.
Nurse shortage	/	Perceived barriers: Insufficient nursing staff to support evidence implementation	M2: The most pressing issue right now is that we may not be able to spare additional instructors for this mentoring program. We’re already understaffed clinically, and we’re concerned this will compromise implementation outcomes. M3: Implementing this fully is quite labor-intensive, but with our department currently understaffed, it’s genuinely challenging.
Positive attitude toward evidence application	/	Perceived facilitators: Healthcare workers support and are willing to promote standardized management and application	M2: This area of interventional guidance really needs proper management. I’m definitely willing to manage it—it’s beneficial for patients, and this field remains largely unexplored in clinical practice. M4: Standardized management would definitely be beneficial, and I’d actively support it. Based on your evidence, this approach is already quite mature and advantageous for patients. Personally, I suggest you promote and publicize it more widely so more colleagues can understand its benefits. M8: Standardization is truly necessary now. While we’re doing a lot, it still needs refinement. Implementing it based on evidence also gives us greater peace of mind. Seeing patients successfully use CIC and no longer worry about urination is genuinely heartening. I hope more standardized management can enhance patient outcomes.

CIC: Clean Intermittent Catheterization.

**Table 9:** Healthcare workers’ CIC knowledge, attitude, and behavior scores (*n* = 156).

Dimensions	Average Score of Item (Mean ± SD)
Knowledge	3.3 ± 0.52
Attitude	2.1 ± 0.41
Behavior	2.3 ± 0.72

**Table 10:** Qualitative interview findings on perceived barriers/facilitators at the level of the practical environment.

Theme	Sub-Theme	Facilitator/Barrier	Statements
Current nursing practice content and processes	Lack of standardized management norms and workflows	Perceived barriers: Absence of institutional and procedural support hinders standardized implementation and personnel management	M3: Standardized processes are absolutely essential. Only with processes can we ensure everyone follows procedures to perform standardized, efficient work. Institutional norms are also necessary—otherwise, personnel management becomes impossible. Processes and systems are fundamental. Before implementation, these must be established first. M9: Personally, I really need a standardized process to guide my work. With institutional norms, I can be guided on how to proceed and what to do, avoiding mistakes. Currently, these are absent, making things rather arbitrary.
	Poor health education outcomes	Perceived barriers: Monotonous teaching methods, complex content, insufficient time, and lack of checklist support lead to inadequate patient comprehension and retention	P2: You speak too fast, cover too much material, and the clinic is so noisy—I barely understood anything. After finishing, you just handed me a sheet of paper to read at home. I didn’t grasp it at all, and even when I tried to read it later, I couldn’t make sense of it. P8: The instructor covered a lot. When asked if I understood, I felt I did, but by the time I got home, I’d forgotten most of it. The materials provided had tiny print and too much text. Some parts were unclear, and I couldn’t ask questions. Worst of all, after just one round of listening and reading, I was expected to do it myself. I was really scared. M8: The time allocated for health education is extremely limited. I feel I couldn’t even provide them with a complete, systematic health education session. I just covered the key points and told them to review the materials carefully at home… Questions? Patients have so many questions, but there’s simply no time to answer them all. M9: When it’s not busy, I might explain more. But when things get hectic, it’s all about explaining while doing. There’s barely enough time to let patients practice once so I can check. I feel like I might have missed a point or two sometimes. It would be great to have a checklist to follow for education—then patients could also check with us where we still need to explain… Right now, I mainly explain verbally while demonstrating the steps. Then I give them a sheet to understand the procedure. Oh, and there’s also an instructional video they can watch later to follow along.
	Lack of follow-up	Perceived barriers: Absence of a systematic follow-up mechanism hinders implementation effectiveness evaluation and continuous improvement	P5: Well, they never followed up with me afterward. Whenever I encountered issues midway, I didn’t know who to ask. P7: I just feel there was no follow-up. When I came back for my follow-up clinic visit, they asked me then, but they never checked in while I was at home. M8: After teaching patients, we don’t consistently track how they manage at home. We’re unsure if they’re doing it correctly, if it’s effective, or if they’re getting follow-up tests… But this is actually essential. If possible, timely follow-up should be conducted.
Practical atmosphere	Physician support	Perceived facilitators: Physicians play a crucial role in preoperative education and decision-making of the patients, enhancing patient acceptance and facilitating the smooth implementation of evidence-based practices	M3: As long as physicians discuss the possibility of CIC post-surgery with patients right at the beginning of preoperative counseling, our subsequent intervention goes very smoothly. Patient acceptance is high—much higher than when nurses or educators directly explain CIC to patients. M7: The main issue now is that some physicians still don’t fully endorse CIC. If all physicians were on board, clinical implementation would be much smoother. Yes, gaining physician support is essential first. P12: I actually didn’t know what CIC was at first. But when the doctor told me I needed it to avoid wearing a urinary bag, I thought, ‘The doctor is so authoritative—they must be looking out for my best interests,’ so I agreed.
	Leadership support	Perceived facilitators: Leadership-driven initiatives and institutional mechanisms enhance implementation effectiveness and organizational responsiveness	M6: Our head nurse has always been eager to advance the management of intermediate care. If leadership mandated training, everyone would definitely participate and implement it. M11: First and foremost, leadership support is essential. Leadership must take the lead in driving progress; otherwise, it simply won’t gain traction. For instance, when the head nurse requires implementation, the nurses under her will follow suit.
	Learning organization	Perceived facilitators: A supportive learning environment and ongoing training mechanisms facilitate the adoption and dissemination of evidence-based practices	M7: Our department’s instructors all emphasize the importance of learning. In our profession, you never stop learning. The department regularly offers training sessions, and everyone is eager to improve their skills. M9: Whenever our department introduces new technologies or launches projects, everyone actively studies first before implementing them. This includes specialized nursing programs and advanced training. We all understand that continuous learning is essential to gain more qualifications and technical skills.
Economic factors	Increasing income	Perceived facilitators: Incorporating practical work into performance evaluations or income distribution can boost participation motivation	M2: If you want people to do these tasks, you have to show them the benefits. It needs to be reflected in their performance evaluations for them to be more willing to do it. Or else, count it as work time—they shouldn’t do it for nothing. M8: If doing these tasks could increase my income, I’d definitely be more willing. I see it as recognition for my hard work.
	Out-of-pocket material purchases	Perceived barriers: Patients must purchase materials at their own expense, with financial burden impacting sustained implementation	P1: Even though a catheter only costs a few bucks each, you need several every day. You never know how long the catheterization will last, and they can’t be reused. When you add it all up, it really gets expensive. If it ends up being too costly, many people might just give up. P11: I know some other hospitals cover catheter costs, but yours doesn’t. Paying out-of-pocket is manageable for a while, but it’s unsustainable long-term.
	Limited funding for implementation	Perceived barriers: Lack of financial support for producing and promoting health education materials	M1: Producing health education materials requires funding. High-quality color printing, design work, and videos all come at a high cost—not to mention whether we have the energy and capability to handle these tasks.
Talent development	Dedicated position	Perceived facilitators: Assigning experienced personnel to manage promotion and oversight facilitates high-quality implementation	M2: It’s best to assign a faculty member exclusively to this task. I believe this is the only way to ensure effective progress. Faculty members who are also engaged in clinical work may struggle to juggle both responsibilities, potentially compromising quality. M3: You need someone dedicated to promotion and management, but this person should ideally have prior teaching experience. CN0 and CN1 staff are definitely not suitable. It would be better to add this dedicated position later, once all processes are standardized.
	Knowledge and skills training and assessment	Perceived facilitators: Implementing systematic training and regular assessments facilitates standardized implementation	M4: Many of us aren’t very familiar with this interim guidance. You probably need to start by training everyone first. Understanding it is the first step toward acceptance, right? M10: Only through training—and intensifying training efforts—can everyone quickly master the relevant knowledge and procedures. Simultaneously, assessments are essential. Regular training and assessments are necessary. Only after passing the assessment can one conduct interventional procedures. If the assessment is failed, then unfortunately, you cannot proceed and will lose the opportunity. This is especially critical for instructors in interventional clinics; strengthening training and assessment rigor is paramount.
	Nurse career development	Perceived facilitators: Providing clear career pathways enhances motivation for sustained engagement	M1: Since the teachers have already put in so much work, if we could build on that foundation to outline a development path for them, they might be more motivated to keep at it. Frankly, it’s about giving them something to look forward to. Right now, so many senior teachers desperately need clear directions for growth. M8: I’m getting older now. If I can’t keep up with clinical work in the future, I need to consider my career path. This seems like a great direction. If I could specialize in this area later, that would be ideal—so I need to master it now.
Patient factors	Inadequate knowledge, attitudes, and confidence levels	Perceived barriers: Insufficient knowledge leads to negative attitudes and low confidence, hindering behavioral implementation	P3: We’re not professionals. We’ve received too much new information, and some points still feel unclear—we’re unsure how to proceed or what to watch out for. We’re afraid of causing side effects if we don’t do it right. P10: After hearing the doctor and nurse explain it, I thought CIC sounded good. But they didn’t train me well enough. I feel like I don’t fully understand it, and I’m nervous when I do it. I’m not sure if I’m doing it right. M9: When patients come to my clinic for home-based guidance or follow-ups, I often find they’re unclear about procedures. Many don’t follow instructions correctly at home. When I remind them we’ve covered this before, some say they forgot, others claim they didn’t understand, and some admit they were afraid to try. This significantly impacts treatment outcomes.
	Poor acceptance	Perceived barriers: Patients exhibit psychological resistance and self-doubt regarding self-catheterization	P1: When they first told me I’d have to insert the catheter myself, I was stunned. I said, ‘What? Me? I’m not a nurse—how could I possibly do that? P7: My immediate reaction was, ‘I can’t do this.’ inserting a catheter is so intimidating—it’s always been the nurses’ job… Now they want me to do it myself? I’ve never been trained, and I have to drink water as instructed? It feels downright absurd—way too hard for me. M6: Whenever we explain CIC to patients, their first reaction is almost always, ‘I have to do it myself? I can’t manage that…’ It’s not just our patients—even some doctors or nurses from other departments were skeptical at first, convinced patients couldn’t handle it themselves.
	Poor adherence and self-management levels	Perceived barriers: Poor patient adherence and inadequate self-management during CIC are factors that hinder the clinical application of evidence	P2: It’s not that I don’t want to follow your instructions—it’s just tough. Like when I have to go out, I can’t stick to the schedule. P4: Honestly, the home care instructions are a bit demanding… It’s not something I can manage just because I want to. M8: Many patients show poor outcomes. When we assess them, we find they haven’t followed the requirements—either the catheterization frequency is wrong or their fluid intake is off… One patient even developed hematuria but still didn’t come to the hospital.
	Significant operational difficulty	Perceived barriers: Patients face significant difficulties in performing CIC, which hinders its clinical application	P5: I don’t know if it’s my technique, but every time I insert the catheter it hurts a bit. Sometimes it hurts so badly I don’t even want to do it anymore. P9: Once after inserting the catheter, I started bleeding. It scared me so much I rushed to the hospital, worried I’d damaged my urethra. P12: We’ve been doing CIC for quite some time now, but sometimes I still can’t get it in. I don’t know why. I’m afraid to push too hard, and it just feels uncomfortable.
	Social support	Perceived facilitators: Social support is a factor that promotes the clinical application of evidence-based practices, such as the companionship and support of family and friends, as well as the advice and support provided by professional healthcare institutions	M6: Home-based monitoring simply wouldn’t work without family assistance, especially at the beginning… Sometimes family members even need to operate the device. M11: Some older patients without family supervision easily make mistakes. P7: I refused at first… But my mom heard the doctor say it was better for me, so she persuaded me to try it. P11: Without my husband reminding me daily, I probably wouldn’t have stuck with it.
	Patient concerns	Perceived barriers: Abandoning the procedure due to worries about operational risks and safety	P6: When I first learned about CIC, I told the doctor I’d never done it before. Inserting a catheter scares me—I wouldn’t know how to handle any complications. P7: My main worry is… Fearing I won’t have the energy to handle catheterization. M8: Many patients have various concerns… We can genuinely sense their anxiety. M10: Expressing concerns is the most common reaction among patients encountering CIC for the first time… Many ultimately choose not to proceed due to these worries.
	Lack of suitable conditions for CIC performance, inconvenient to execute	Perceived barriers: Lack of privacy and clean environments limit implementation	P4: It’s very inconvenient to go out for long periods… Public restrooms aren’t very clean, and I worry about infections (urinary tract infection), so I try to avoid catheterization whenever possible. P8: Catheterization outside is so inconvenient—restroom stalls don’t have mirrors or handwashing facilities… It would be great if future restrooms could be designed with catheterization stations like at home.
	Image and comfort requirements	Perceived facilitators: Avoiding continuous wear of urine bags, improving quality of life and image comfort	P2: Not having to constantly wear a urine bag is definitely better… It’s embarrassing when you can’t hide it. P10: With the urine bag always on me, I feel like I’ve become smelly… I always sense a urine odor on my body. P12: Having the catheter inserted for days after surgery was extremely uncomfortable…

CIC: Clean Intermittent Catheterization; CN0: Nurses with less than one year of experience; CN1: Nurses with 1 to 4 years of experience.

**Table 11:** Patients’ CIC knowledge, attitudes, and practices scores (*n* = 300).

Dimensions	Average Score of Item (Mean ± SD)
Knowledge	3.4 ± 0.69
Attitude	2.8 ± 0.35
Behavior	2.8 ± 1.06

**Table 12:** Patients’ overall acceptance scores for CIC and scores across each dimension (*n* = 300).

Dimensions	Total Scores	Average Score (Mean ± SD)
Fear	25	14.8 ± 5.39
Self-esteem	40	21.8 ± 4.47
Acceptance	5	2.1 ± 1.16
Total score	70	38.6 ± 6.92

**Table 13:** Patient CIC implementation difficulty scores (*n* = 300).

Dimensions	Total Scores	Average Score (Mean ± SD)
Difficulty frequency	39	21.5 ± 5.26
Difficulty severity	39	20.8 ± 5.15

**Table 14:** Patient CIC self-management total score and scores across dimensions (*n* = 300).

Dimensions	Average Score of Item (Mean ± SD)	Score Ranking
Disease symptom management	3.2 ± 0.61	2
Daily living activities management	4.1 ± 0.45	1
Intermittent catheterization behavior management	3.2 ± 0.93	3
Emotional management and social reintegration	2.9 ± 0.70	4
Total score	123.0 ± 20.65	/

**Table 15:** Integrated results of mixed studies.

KTA Domain	Qualitative Theme	Quantitative Indicator	Convergence/Divergence	Implementation Implication
evidence itself	/	Limited operational clarity and usability of evidence (Table 7)	Convergence	“Limited operational clarity and usability of evidence,” as reflected in the interviews, should be considered in conjunction with the attitude and behavior scores
	/	Meeting patient needs (Table 7)	Divergence	needs identified in the interviews should be combined with the attitude scores
	/	Meeting healthcare workers’ needs (Table 7)	Divergence	needs identified in the interviews should be combined with the attitude scores
potential adopters	Suboptimal knowledge, attitudes, and behaviors among healthcare staff	Limited CIC knowledge base (Table 8)	Convergence	limited knowledge of CIC revealed in the interviews should be combined with the CISC-KAPS knowledge scores
	/	Limited evidence-based practice knowledge (Table 8)	Convergence	limited knowledge of evidence-based practice revealed in the interviews should be combined with the CISC-KAPS behavioral scores
	Suboptimal knowledge, attitudes, and behaviors among healthcare staff	Insufficient CIC-related skills (Table 8)	Convergence	insufficient CIC-related skills identified in the interviews should be combined with the CISC-KAPS behavioral scores
	/	Low willingness among healthcare staff to promote and adopt the practice (Table 8)	Convergence	low willingness indicated in the interviews should be combined with the CISC-KAPS attitude scores
	/	Insufficient autonomy in (Table 8) practice	Convergence	findings regarding insufficient autonomy in practice from the interviews should be combined with the CISC-KAPS attitude scores
	/	Lack of recognition regarding task importance (Table 8)	Convergence	lack of awareness of importance reflected in the interviews should be linked to the lower CISC-KAPS attitude scores
	/	Perceived risk and liability concerns (Table 8)	Convergence	healthcare workers’ uncertainty should be linked with KAP attitude/behavior scores
	/	Increased workload (Table 8)	Convergence	healthcare workers’ uncertainty should be linked with KAP attitude/behavior scores
	/	Nurse shortage (Table 8)	Convergence	shortage of nursing staff reported in the interviews should be linked to the low CISC-KAPS attitude scores
	/	Positive attitude toward evidence application (Table 8)	Divergence	positive attitude toward the use of evidence reflected in the interviews should be reconciled with the low attitude score
practical environment	/	Lack of standardized management norms and workflows (Table 10)	Convergence	lack of standardized management guidelines and workflows identified in the interviews should be linked to the low behavioral scores
	/	Poor health education outcomes (Table 10)	Convergence	poor health education from interviews should be linked with low behavior, confidence and adherence scores
	/	Lack of follow-up (Table 10)	Convergence	lack of follow-up from interviews should be linked with low behavior scores
	Suboptimal levels of patient knowledge, attitudes, and behaviors	Physician support (Table 10)	Divergence	doctors’ support as reflected in the interviews should be linked to their and patients’ attitude scores
	/	Leadership support (Table 10)	Divergence	leadership support identified in the interviews should be linked to the scores of attitudes and behaviors
	/	Learning organization (Table 10)	Divergence	discrepancy between the learning organization described in the interviews and the low knowledge scores should be addressed
	/	Increasing income (Table 10)	Divergence	a discrepancy between the increase in business revenue in the interview findings and the low attitude scores
	/	Out-of-pocket material purchases (Table 10)	Convergence	out-of-pocket material purchases mentioned in the interviews should be considered in conjunction with the low attitude, adherence and acceptance scores
	/	Limited funding for implementation (Table 10)	Convergence	limited funding for implementation mentioned in the interviews should be considered in conjunction with the low attitude scores
	/	Dedicated position (Table 10)	Divergence	findings regarding specialized position management from the interviews should be linked to the KAP attitude/behavior scores
	Suboptimal levels of patient knowledge, attitudes, and behaviors	Knowledge and skills training and assessment (Table 10)	Divergence	training and assessments discussed in the interviews should be linked to the CISC-KAPS scores
	/	Nurse career development (Table 10)	Divergence	nurses’ career development from the interviews should be linked to their KAP attitude scores
	Suboptimal levels of patient knowledge, attitudes, and behaviors; Poor patient confidence in performing CIC	Inadequate knowledge, attitudes, and moderate confidence levels (Table 10)	Convergence	low patient confidence from interviews should be integrated with SCSCISC scores; Inadequate level of patient knowledge and attitude reflected in the interviews should be evaluated in conjunction with the CISC-KAPS score.
	Poor patient acceptance of CIC	Inadequate acceptance (Table 10)	Convergence	low patient acceptance reported in the interviews should be considered in conjunction with the ICAT score
	Poor patient adherence to CIC; Patient CIC-related self-management levels were inadequate	Poor adherence and self-management levels (Table 10)	Convergence	poor patient adherence observed in the interviews should be evaluated in conjunction with the ICAS score; low level of patient self-management reflected in the interviews should be evaluated in conjunction with the CIC Self-Management Questionnaire scores.
	Significant difficulties in implementing CIC for patients	Significant operational difficulty (Table 10)	Convergence	significant difficulties patients reported in the interview should be considered in conjunction with the ICDQ score
	/	Social support (Table 10)	Convergence	patient’s social support should be linked to their KAP attitude/behavior scores
	/	Patient concerns (Table 10)	Convergence	patient’s social support should be linked to their KAP attitude/behavior and acceptance scores
	/	Lack of suitable conditions for CIC performance, inconvenient to execute (Table 10)	Convergence	patients’ reports of a lack of conditions for implementation and difficulties in execution should be linked to their low behavioral and adherence scores
	/	Image and comfort requirements (Table 10)	Divergence	discrepancies between the image and comfort preferences reported by patients and their low attitude and acceptance scores

CIC: Clean Intermittent Catheterization.

## Data Availability

The data presented in this study are available on request from the corresponding author due to the privacy protection of the participants.
